# Embodied cognition driven Qigong: a cross-sectional study and a pilot randomized controlled trial on managing depression and preventing relapse in substance dependence

**DOI:** 10.3389/fpubh.2024.1388887

**Published:** 2024-11-01

**Authors:** Chao Sun, Siyao Yang, Xiaojun Wang, Yongcong Shao, Xuetong Huang, Huanhuan Qi, Zhuolin Zhang, Guobai Su

**Affiliations:** ^1^School of Psychology, Beijing Sport University, Beijing, China; ^2^Tsinghua International School, Beijing, China; ^3^China Wushu School, Beijing Sport University, Beijing, China; ^4^Department of Physical Education, Tianjin Medical University, Tianjin, China

**Keywords:** depression, recurrence, embodied cognition, Qigong, substance-related disorders

## Abstract

**Objective:**

Substance use disorders (SUDs) pose a significant challenge to public health systems worldwide, with persistent high relapse rates underscoring the urgency for innovative intervention strategies. This study embarked on a systematic exploration of the factors influencing relapse among individuals with SUDs, alongside the implementation of targeted Health Qigong intervention to mitigate these effects based on the embodied cognition.

**Methods:**

In the cross-sectional survey, a total of 398 male substance users were recruited to complete the questionnaires assessing depression, self-esteem, resilience, and relapse inclination. Subsequently, 60 participants diagnosed with depression were selected for a Pilot RCT, randomly divided into an intervention group, which underwent a 12-week Health Qigong program designed to combat depression through embodied cognition principles, and a control group, which maintained their daily routines.

**Results:**

Structural equation modeling demonstrated that depression not only had a direct effect on substance users’ relapse, but also exerted an indirect effect through three paths: firstly, via the mediating role of self-esteem; secondly, via the mediating role of resilience; and thirdly, via the chain-mediated role of self-esteem and resilience. Two-factor mixed-design ANOVA showed that the 12-week targeted Health Qigong training not only mitigated depression in individuals with SUDs, but also enhanced self-esteem and mental resilience, and reducing the propensity for relapse.

**Conclusion:**

This research identifies critical factors influencing relapse in individuals with SUDs and introduces a novel, non-pharmacological intervention that significantly diminishes relapse rates and enhances recovery outcomes. It highlights the importance of physical activity in promoting mental health improvement and integrates traditional Chinese exercises with contemporary psychological insights, offering a comprehensive approach to drug rehabilitation and the integration of cultural practices in holistic health interventions.

## 1 Introduction

Substance use disorders (SUDs) represent a significant public health issue worldwide, stemming from complex brain disorders linked to recreational drug use (1). These disorders impact both physical and mental health and place a substantial strain on economies and societies (2, 3). The World Drug Report 2022 indicates that around 284 million people aged 15–64 engage in drug use globally, showing a 26% increase over the past decade. Despite concerted efforts in drug rehabilitation worldwide, the high rates of relapse, defined as returning to drug use after a period of abstinence (4), present a persistent challenge. Alarmingly, relapse rates reach 80–90% in certain areas, with China experiencing rates over 90% (5). This scenario underscores the urgent need for research into the determinants and mechanisms of relapse to develop more efficacious relapse prevention strategies in addiction recovery.

### 1.1 Depression and relapse

Depression is commonly observed alongside SUDs, demonstrating formidable obstacles to recovery and sustained abstinence (6). Neuroscientific research has uncovered neurobiological similarities in brain structure and function between SUDs and depression, suggesting shared pathophysiological underpinnings (7, 8). Epidemiological studies further highlight a robust association between SUDs and mental health disorders. Individuals suffering from severe depression are nearly twice as likely to develop SUDs as the general population (9, 10). Additionally, SUDs diagnoses are associated with a three to fourfold escalation in depression rates (11), a consistent pattern across substances like nicotine, alcohol, cannabis, opioids, and psychostimulants (9, 10, 12). A recent longitudinal study over 2 years involving 3,320 college-aged females found a significant association between alcohol and cannabis use and increased depression scores (13). The self-medication hypothesis suggests that individuals may resort to substances to mitigate negative emotions, such as depression and anxiety, potentially leading to substance dependence (14). Evidently, the severity of depression in individuals with SUDs correlates positively with their likelihood of relapse.

### 1.2 The mediating role of self-esteem and resilience

Self-esteem, integral to psychological capital and self-concept, is an individual’s consistent self-evaluation of worth, evident in levels of self-acceptance or rejection. Shahar and Davidson (15) argue that depression, a significant stressor, can erode self-concept and reduce self-esteem. Empirical evidence from longitudinal studies confirms this, showing that depressive states lead to declines in self-esteem among adolescents and university students (16, 17). Furthermore, using the experience sampling method, researchers assessed momentary self-esteem levels in individuals with clinical major depression and healthy controls. This assessment, conducted at 10 intervals daily over 6 days, revealed a pronounced double inverted U-shaped pattern of self-esteem in the depressed group, significantly lower than that of the controls, highlighting the profound impact of depression on self-esteem (18). Mruk (19) underscores the importance of self-esteem as a buffer against stress, vital for overcoming challenges in drug rehabilitation, such as withdrawal symptoms and relapse risk. This relationship is supported by a negative correlation between self-esteem and drug cravings, and a positive correlation with rehabilitation success (20). Further research indicates that low self-esteem may drive individuals toward substance use as a way to manage emotions (21, 22). Consequently, this research proposes Hypothesis 1 (H1): Self-esteem mediates the relationship between depressive mood and the propensity for relapse in individuals with SUDs.

Psychological resilience, an adaptive process, capacity, or characteristic, equips individuals to navigate through adversities, traumas, and significant stressors effectively (23, 24). Depressive individuals often exhibit diminished reactivity to both positive and negative emotions, leading to rigid coping strategies for stress and negative affect. This rigidity typically results in lower levels of psychological resilience (25). Vesco et al. (26) similarly identified the detrimental effects of negative thinking patterns on psychological resilience. Resilience not only facilitates emotional regulation and tolerance of negative emotions but also acts as a deterrent against the development of substance dependence (27). A cross-sectional study indicates that higher resilience levels correlate with decreased tendencies toward smoking, nicotine, alcohol consumption, and substance use (28). Therefore, this study hypothesizes (H2) that psychological resilience mediates the impact of depressive emotions on relapse in individuals with SUDs.

Self-esteem and resilience, as intertwined psychological protective factors, play an essential role in mitigating anxiety and depression amid setbacks and challenges (29). Connell et al. (30) propose that a positive self-concept is crucial for the development of psychological resilience. Research by Masten and her team further supports this, identifying positive self-evaluations such as self-confidence, high self-esteem, and self-efficacy as key characteristics that foster resilience (31). Particularly, high self-esteem boosts confidence in one’s abilities and promotes proactive strategies, thereby enhancing resilience. This relationship is underscored by a meta-analysis which reported a significant correlation (*r* = 0.55) between self-esteem and resilience (32). Moreover, research has shown that self-esteem and resilience sequentially mediate adaptability in the face of adversity (33). Therefore, these findings collectively suggest that self-esteem not only influences resilience but also plays a critical role in individuals’ ability to adapt and recover, leading to hypothesis 3 (H3) that in individuals with SUDs, depression impacts relapse through the chain-mediated effects of self-esteem and resilience.

### 1.3 Intervention effect of health Qigong

Interventions aimed at depression, which effectively mitigate substance use, are associated with improved symptoms, reduced relapse rates, and enhanced recovery outcomes (34). Current treatments, including pharmacotherapy, cognitive-behavioral therapy (CBT), and psychotherapy, have shown efficacy in managing depressive symptoms in individuals with SUDs. However, these approaches are not without their limitations. For example, pharmacotherapy often faces challenges such as high costs and the potential for dependency, while CBT and psychotherapy may suffer from low patient adherence and inadequate evaluation techniques (35). In contrast, physical exercise not only addresses these limitations but also reverses physiological and neural damage caused by substance abuse and increases the release of brain-derived neurotrophic factor (BDNF), contributing to its antidepressant effects (36). Furthermore, research including a systematic review and meta-analysis has suggested that Qigong practice may alleviate depressive symptoms in individuals with SUDs, though the limited number and low methodological quality of these studies necessitate cautious interpretation (37). Consequently, further high-quality randomized controlled trials are needed to confirm these findings. Notably, previous investigations into the mental health benefits of Qigong have often overlooked the significant role of body posture, which may influence its therapeutic effectiveness.

The embodied cognition theory posits a fundamental and essential connection between cognitive processes and the physical body, suggesting that cognition is derived from bodily experiences and interactions. According to this perspective, cognitive functions and thought patterns are shaped by various factors, including anatomical structures, movement dynamics, sensory feedback, and the experiential qualities of movement (38). Recognizing the interconnectedness of physical and mental health, it can be inferred that altering bodily movements can significantly enhance psychological well-being and contribute to the management of various mental disorders, such as depression, insomnia, and autism (39). Darwin’s observations highlighted that slumped, relaxed, and downward postures are indicative of depression and sadness, whereas erect, upright, and open postures correlate with positive emotions (40). These emotional manifestations are evident not only in static postures but also in dynamic movements, such as walking patterns. Michalak et al. (41) found significant differences in the gait of individuals with severe depression compared to healthy individuals, with the former showing slower walking speeds, diminished arm swings, decreased vertical head motions, and more slouched postures. Furthermore, individuals experiencing depression displayed shorter stride lengths and increased stride durations relative to the healthy control group (42). Health Qigong, characterized by its static and dynamic body postures, represents a unique aspect of physical exercise (43). Static Qigong entails such as sitting meditation or stationary poses, focusing on breath control, body relaxation, concentrated attention, and the integration of mind and body to achieve physical health, increased internal energy, and mental serenity. Dynamic Qigong, conversely, emphasizes active bodily adjustments, breath guidance, and the application of principles to improve flexibility and strength, thereby promoting the efficient flow of qi and blood.

The embodied cognition theory emphasizes the interplay between mood and physical movement, suggesting that adjustments in body posture and movement can influence emotional states. Depression often presents with physical symptoms such as slowed movement, slumped posture, muscle tension, and pain (44, 45). To tackle these physical manifestations of depression, we meticulously curated Health Qigong exercises from traditional Chinese practices known for improving posture and promoting chest expansion. These include Baduanjin, Five Animal Exercises, 12-form Daoyin Health Exercises, and Mawangdui Daoyin. We developed a comprehensive anti-depression exercise regimen that features a sequence of movements such as the ready form, Holding Sky with Hands, Shuangyu Xuange, Longdeng, Tiger Pounce, Rouji Style, Deer Running, Golden Rooster Dawn, Bird Flying, Returning Qi to the Source, and the concluding form. Comprehensive descriptions of each action within the program can be found in Supplementary Table S1. Therefore, hypothesis 4 (H4) postulates that sustained engagement in Health Qigong not only diminishes depression among individuals with SUDs but also boosts self-esteem and resilience, and lower the risk of relapse.

### 1.4 The current study

Despite extensive research into the comorbidity of depression and SUDs and their relapse, significant gaps remain. Although the link between depression and SUDs relapse is acknowledged, the mechanisms underlying their interaction are still unclear. Additionally, while physical activity interventions have shown potential to alleviate depression symptoms, studies that target depression to subsequently reduce substance use relapse are scarce and often methodologically weak. Addressing these research needs, our study began with a cross-sectional analysis exploring the relationship between depression and SUDs relapse, with a focus on the potential mediating roles of self-esteem and psychological resilience. Following this, we conducted a pilot randomized controlled trial (RCT) grounded in embodied cognition theory. This trial utilized Health Qigong interventions aimed specifically at the physical manifestations of depression, assessing their effectiveness in improving depressive symptoms, enhancing self-esteem and psychological resilience, and reducing relapse risk. These two studies collectively aim to provide new empirical insights into the factors influencing SUDs relapse and their treatment, thereby establishing a scientific basis for developing innovative psychosocial intervention strategies.

## 2 Study 1: cross-sectional analysis of depression, self-esteem, resilience, and relapse in individuals with SUDs

### 2.1 Purpose

This study aims to explore the relationship between depression and relapse in patients with SUDs, and to further examine the roles of self-esteem and psychological resilience as both individual and sequential mediators in this relationship.

### 2.2 Methods

#### 2.2.1 Participants

The study recruited 420 male substance users from rehabilitation facilities in Guangxi, China, employing convenience sampling. A group-administered paper questionnaire was distributed and collected on-site for the survey. The final analysis included a total of 398 valid questionnaires, yielding a response rate of approximately 94.76% after excluding any incomplete or inaccurate submissions. The participants’ mean age was 38.95 ± 8.04 years, with an average drug use duration of 4.53 ± 1.66 years. The detailed demographic characteristics of the participants were summarized in Table 1.

**Table 1:** Descriptives of the sample.

Variable	Cross-sectional surveys	Pilot RCT			
	*N*	Intervention group	Control group	*χ* ^2^	*p*
Education					
Elementary	166	14	11	0.46	0.49
Junior	218	15	17		
Senior	14	0	0		
Marriage					
Single	202	15	15	0.32	0.85
Married	130	10	8		
Divorce	66	4	5		
Drug abuse pattern					
Snorting	223	16	11	2.85	0.42
Intramuscular injection	4	1	0		
Intravenous injection	111	7	9		
Combination	60	5	8		
Relapse					
Yes	346	26	26	1.23	0.27
No	52	3	2		

#### 2.2.2 Measures

##### 2.2.2.1 The self-rating depression scale (SDS)

The SDS is widely utilized in both clinical and research domains to assess the severity of depressive symptoms, monitor symptom changes over time, and evaluate the efficacy of interventions or therapies of depression (46). It consists of 20 items, employing a four-point Likert-type response format scale that ranges from “1” (not at all) to “4” (very much) to indicate the severity of depressive symptoms. Calculation of the SDS raw score entails summing the scores of the 20 items, which is then adjusted by multiplying it with a coefficient of 1.25 and rounding to the nearest whole number to derive the standard score. Higher scores on the SDS indicate greater severity of depressive symptoms, whereas lower scores suggest fewer or milder symptoms. Specifically, a score below 53 indicates no depressive symptoms, 53–62 suggests mild symptoms, 63–72 indicates moderate symptoms, and a score of 72 or above signifies severe depression. In this study, the SDS demonstrated good reliability, with a Cronbach’s alpha value of 0.86.

##### 2.2.2.2 Self-esteem scale (SES)

The SES is designed to assess an individual’s self-esteem or self-worth (47). It comprised 10 items, requiring participants to express their level of agreement using a Likert-type scale that ranges from “1” (strongly disagree) to “4” (strongly agree). Higher scores on this scale are indicative of greater self-esteem, signifying strong self-acceptance and a positive self-perception. On the other hand, lower scores suggest reduced self-esteem, highlighting potential areas where personal growth and development may be necessary. The reliability of the scale in this study is supported by a Cronbach’s alpha value of 0.89, indicating high internal consistency.

##### 2.2.2.3 10-item Connor–Davidson resilience scale (CD-RISC-10)

This scale is a tool designed to measure an individual’s resilience, providing an approach to understand their capacity to effectively cope with stress, adversity, and various life challenges (21). It comprises 10 items, each rated on a 5-point scale ranging from 0 (“never”) to 4 (“always”). Generally, elevated scores on the CD-RISC-10 are indicative of greater resilience, showcasing a person’s capacity to recover from difficulties, sustain psychological well-being, and adapt successfully to stressful circumstances. The scale’s reliability in this study is evidenced by a Cronbach’s alpha value of 0.91, indicating a high degree of internal consistency.

##### 2.2.2.4 Relapse inclination questionnaire (RIQ)

This scale serves as an instrument designed to evaluate the likelihood of individuals relapsing onto substance use after completing drug treatment (48). It delineates five key dimensions: confidence in abstaining from drugs, perceived impact of drug use, influence of the surrounding environment, degree of physical and mental harm experience, and the availability of a support system. The RIQ consists of 18 items, with responses gathered using a six-point Likert scale ranging from 0 (least severe) to 5 (most severe). A higher score on this scale suggests a higher probability of relapse. The reliability of the RIQ in this study is substantiated by a Cronbach’s alpha value of 0.87, indicating good internal consistency.

#### 2.2.3 Statistical analysis

Data analysis was initially conducted using SPSS 22.0, employing the Harman single-factor test to assess potential common method bias, a concern when questionnaires are used to measure all variables. Subsequent descriptive analysis and Pearson correlation were performed to examine the relationships among the principal variables. Additionally, AMOS 24.0 was used to analyze structural equation models that investigate the direct effects of depression on relapse among individuals with SUDs, as well as the separate and sequential mediating roles of self-esteem and psychological resilience.

### 2.3 Results

#### 2.3.1 Common method deviation test

The Harman single factor test was used to examine the common method deviation. All the questionnaire items were included in exploratory factor analysis. The first factor accounted for 29.04% of the total variance, which fell below the threshold of 40%, indicating an absence of common method deviation.

#### 2.3.2 Descriptive statistics and correlations of variables

**Figure 1:**
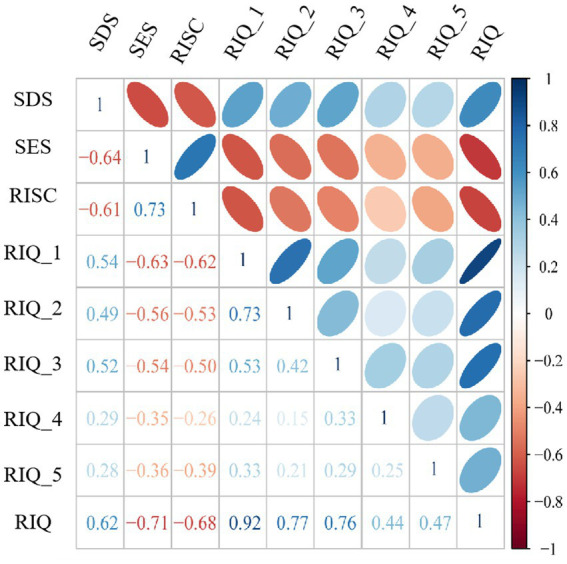
The correlation matrix among variables is being examined.

The mean value and standard deviation (M ± SD) for each variable were as follows: SDS (2.76 ± 0.67), SES (2.69 ± 0.52), CD-RISC-10 (3.20 ± 0.67), RIQ (1.52 ± 0.67). Figure 1 presented the correlation matrix among these variables, which showed that SDS was positively correlated with RIQ (*r* = 0.62, *p* < 0.01) but negatively correlated with both SES (*r* = −0.64, *p* < 0.01) and CD-RISC-10 (*r* = −0.61, *p* < 0.01); conversely, SES was positively correlated with CD-RISC-10 (*r* = 0.73, *p* < 0.01) but negatively correlated with RIQ (*r* = 0.-0.71, *p* < 0.01); in addition, CD-RISC-10 exhibited a negative correlation with RIQ (*r* = −0.68, *p* < 0.01). With this in mind, we can further explore the interplay between the aforementioned factors.

#### 2.3.3 Significance test of the mediating effect

**Figure 2:**
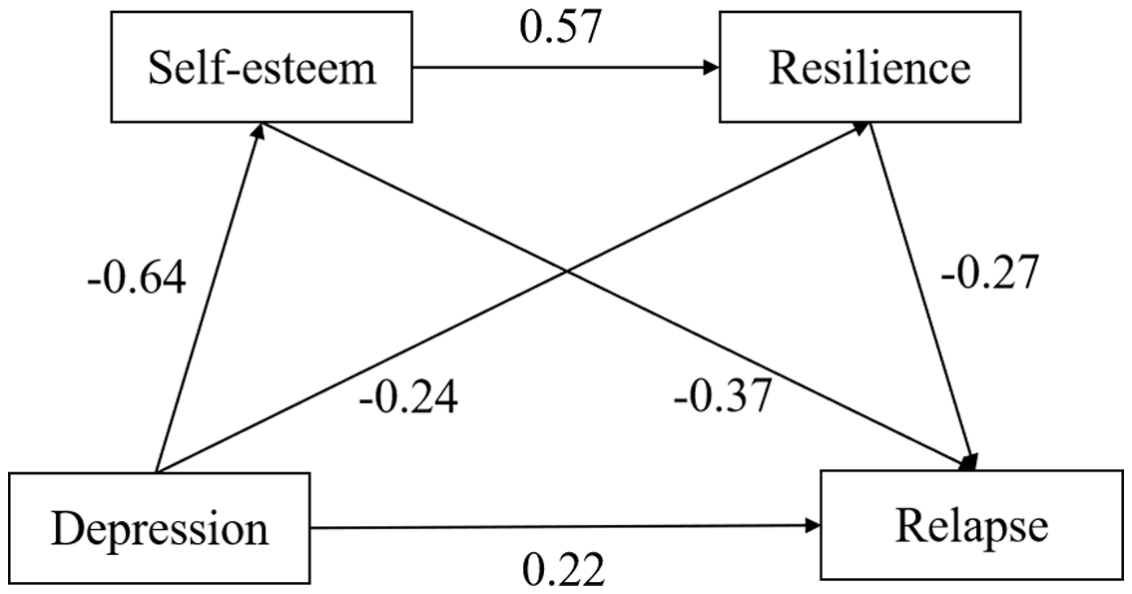
The path diagram of mediating effect of self-esteem and resilience between depression and relapse.

The theoretical model for this study was developed based on our initial hypotheses and the results from correlation analysis, as illustrated in Figure 2. The model demonstrates an optimal fit, with fit indices reported as follows: χ^2^/df = 4.87, CFI = 0.94, TLI = 0.91, RMSEA = 0.04, and SRMR = 0.04 (details in Table 2). Given this robust model fit, we proceeded to perform a Bootstrap analysis using AMOS, with 5,000 resamples to ensure statistical reliability and validity.

**Table 2:** Table model inspection.

Fit indices	Recommended threshold	Scores	Remarks
χ^2^/df	1 < χ^2^/df < 5	4.87	Acceptable
CFI	>0.9	0.94	Acceptable
TLI	>0.9	0.91	Acceptable
RMSEA	<0.08	0.04	Acceptable
SRMR	<0.08	0.04	Acceptable

The results indicated that the direct effect of depression on relapse was 0.22 (*p* < 0.01), with a 95% confidence interval of [0.13, 0.30]. The indirect effects were also significant: depression’s impact on relapse through self-esteem was 0.24 (*p* < 0.01), with a confidence interval of [0.17, 0.31], and through resilience, it was 0.07 (*p* < 0.01), with a confidence interval of [0.03, 0.10]. Furthermore, the sequential mediation pathway from depression to relapse via self-esteem followed by resilience yielded an indirect effect of 0.10 (*p* < 0.01), with a confidence interval of [0.06, 0.15]. The total indirect effect size was 0.40 (*p* < 0.01), with a confidence interval of [0.33, 0.48], demonstrating that both self-esteem and resilience function as mediators, both individually and in sequence, in the relationship between depression and relapse (refer to Table 3).

**Table 3:** Bootstrap test on the indirect effects of depression on relapse.

	Path	Effect value	SE	LLCI	ULCI
Direct effect	Path_1	0.22	0.04	0.13	0.30
Indirect effect	Path_2	0.24	0.03	0.17	0.31
	Path_3	0.07	0.02	0.03	0.10
	Path_4	0.10	0.02	0.06	0.15
Total effect		0.62	0.04	0.54	0.69

LLCI, the lower limit of the confidence interval; ULCI, the upper limit of the confidence interval. Path_1: Depression → relapse; Path_2: Depression → self-esteem → relapse; Path_3: Depression → resilience → relapse; Path_4: Depression → self-esteem → resilience → relapse.

## 3 Study 2: pilot RCT of Qigong intervention for depression management and relapse prevention in substance dependence

### 3.1 Purpose

This study investigates whether a 12-week Health Qigong intervention, aimed at alleviating the physical manifestations of depression, can reduce depressive symptoms in individuals with SUDs. Additionally, it examines whether this intervention can enhance self-esteem and psychological resilience, and subsequently lower the likelihood of relapse.

### 3.2 Method

#### 3.2.1 Participants

In June 2023, a pilot RCT recruited 60 patients diagnosed with moderate to severe depression based on findings from Study I’s cross-sectional survey. The inclusion criteria were as follows: (1) adult males aged between 18 and 60 years; (2) individuals meeting the DSM-5 criteria for SUDs; and (3) participants displaying moderate to severe depressive symptoms, as measured by the SDS, ensuring the study targeted the correct demographic. The exclusion criteria included: (1) patients with psychiatric diagnoses other than depression; (2) individuals with interpersonal communication disorders; and (3) those scheduled to complete drug treatment in isolation within the next 4 months. All 57 eligible participants were randomly assigned to either the intervention or control group using a computer-generated random numbers table for simple randomization. Each participant received a unique identifier that was entered into the randomization software, ensuring equal chances of allocation to one of the two groups. This method maintained balance between the groups and minimized potential selection biases. To preserve the integrity of the allocation process, the random allocation sequence was concealed from the researchers responsible for recruiting participants. Ultimately, following the application of inclusion and exclusion criteria and completing the randomization, the Qigong group consisted of 29 participants, while the control group had 28. There were no significant differences in age [(38.31 ± 5.78) vs. (36.28 ± 4.96), *t* = 1.42, *p* = 0.16] or duration of drug use [(4.79 + 1.82) vs. (4.96 + 1.50), *t* = −0.38, *p* = 0.70] between the intervention and control groups. Additional demographic details for both groups are presented in Table 1. The study was approved by the ethics committee of the drug rehabilitation center. All participants voluntarily took part in the program and provided written informed consent.

#### 3.2.2 Intervention program and implementation of health Qigong

The detailed plan for the 12-week Qigong Intervention Program is presented in Table 4. The program was implemented on the playground of Guangxi rehabilitation facilities, under the guidance of the Health Qigong Ambassador of Guangxi Province and the Director of the Traditional Sports Health Research Center at China Wushu Academy. The interventions were scheduled from 4:00 to 5:00 p.m., four times a week, for a total of 12 weeks.

**Table 4:** Twelve-week Qigong intervention program for depression.

Week	Objectives	Exercises	Frequency
Weeks 1–2	Familiarize with basic movements and adjust breathing	Ready form, Holding Sky with Hands, Shuangyu Xuange, Concluding form	Four times a week, 1 h each session
Weeks 3–4	Strengthen learned movements, increase fluidity	Ready form, Longdeng, Tiger Pounce, Concluding form	
Weeks 5–6	Master more movements, increase training complexity	Ready form, Rouji Style, Deer Running, Concluding form	
Weeks 7–8	Review and consolidate all movements, improve accuracy and coordination	Full cycle of all movements including Golden Rooster Dawn, Bird Flying, Concluding form	
Weeks 9–10	Enhance physical and cardiorespiratory fitness, deepen synchronization of breath and movement	High-intensity cycle of all movements, focus on Returning Qi to the Source, Concluding form	
Weeks 11–12	Integrate all learned content, evaluate effects, prepare for long-term practice	Comprehensive review of all movements, in-depth discussion of effects and feelings, Concluding form	

#### 3.2.3 Procedures

**Figure 3:**
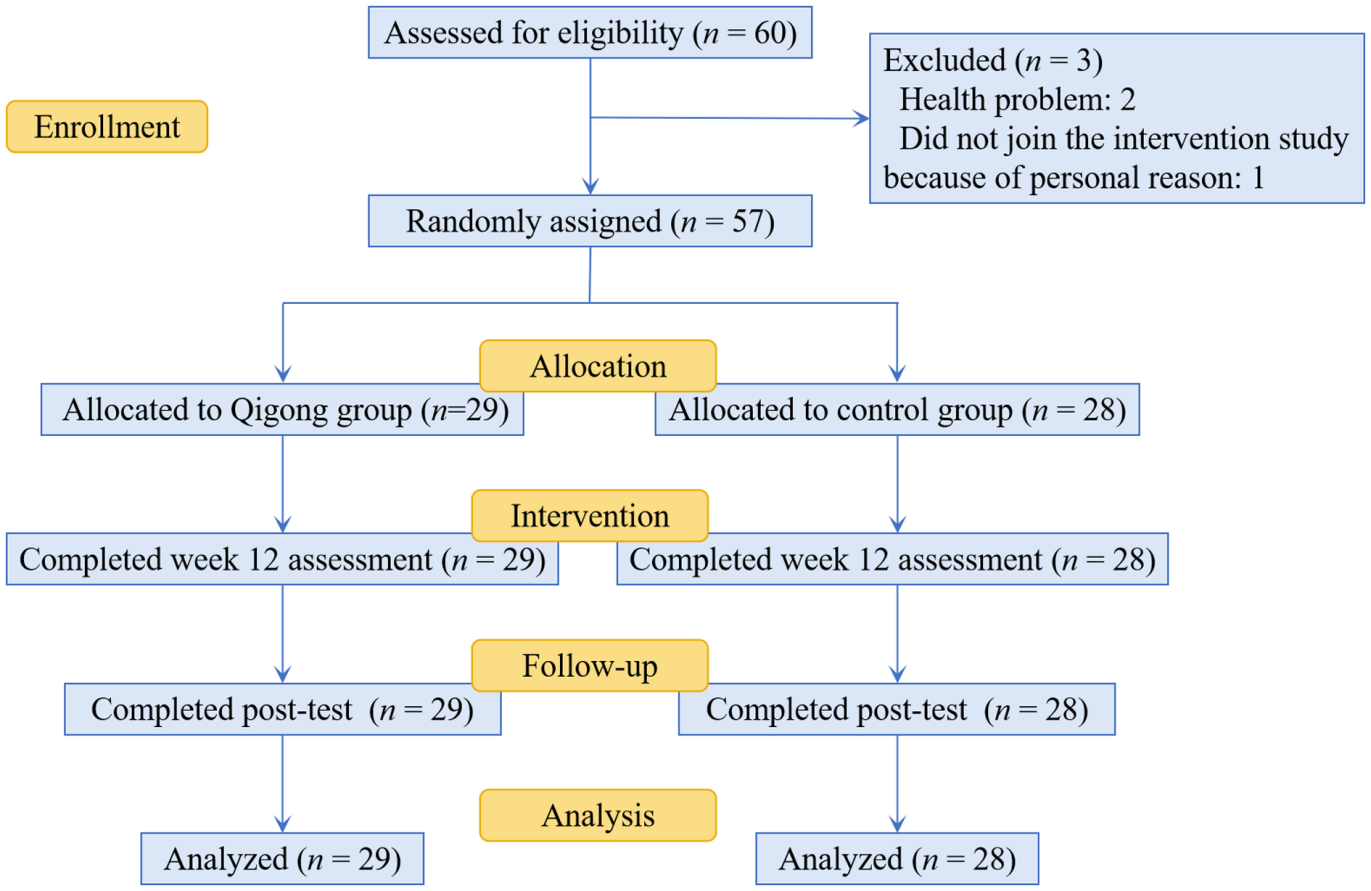
Schematic presentation of the flow of participants through screening, randomization, and the interventions.

Firstly, we obtained baseline scores (pre-test scores) for both the intervention and the control group from the results of Study 1. From July to September 2023, both groups participated in the experimental intervention for a period of 12 weeks. The control group continued their daily routines without any special physical exercise, whereas the intervention group engaged in Health Qigong practice, with the specific intervention plan detailed in Table 4. No additional interventions were introduced during this period. In early October of the same year, upon completion of the 12-week experiment, post-test measurements of the SDS, SES, CD-RISC-10, and RIQ were conducted to evaluate the outcomes. All participants attended each session, resulting in a 100% adherence rate for both groups. The experimental design is detailed in Figure 3.

#### 3.2.4 Statistical analysis

This study utilized SPSS 22.0 for data analysis. Initially, we employed independent samples t-tests and chi-square tests to explore demographic differences between the intervention and control groups. We then conducted descriptive statistics and Shapiro–Wilk (S-W) normality tests on pre-test and post-test data for depression, self-esteem, resilience, and relapse variables, assessing differences in baseline characteristics. Finally, we applied a two-factor mixed-design ANOVA to explore the impact of a 12-week Health Qigong training on depression, self-esteem, psychological resilience, and relapse propensity in individuals with SUDs.

### 3.3 Results

#### 3.3.1 Descriptive statistics and normality tests

Table 5 presents the descriptive statistics for pre-test and post-test data on SDS, SES, CD-RISC-10, and RIQ variables for both the intervention and control groups. Independent samples *T*-tests conducted on the baseline values of these variables indicated no statistically significant differences between the groups (*p* > 0.05). Normality tests on the pre-test and post-test data for the same variables confirmed a normal distribution in both groups (*p* > 0.05). This verification of normality permits subsequent analysis using mixed-design ANOVA.

**Table 5:** Descriptive statistics and normality tests.

Variable	Time	Group	M ± SD	*t* (*p*)	S-W (*p*)
SDS	Pre-test	IG	3.44 ± 0.28	1.35 (0.18)	0.96 (0.24)
		CG	3.35 ± 0.20		0.94 (0.09)
	Post-test	IG	2.56 ± 0.27	\	0.95 (0.23)
		CG	3.22 ± 0.34		0.95 (0.15)
SES	Pre-test	IG	2.11 ± 0.33	−0.91 (0.36)	0.97 (0.51)
		CG	2.19 ± 0.33		0.98 (0.89)
	Post-test	IG	2.76 ± 0.39	\	0.94 (0.08)
		CG	2.35 + 0.40		0.94 (0.14)
CD-RISC-10	Pre-test	IG	2.56 ± 0.30	−0.29 (0.77)	0.94 (0.13)
		CG	2.58 ± 0.31		0.98 (0.85)
	Post-test	IG	3.01 ± 0.50	\	0.96 (0.41)
		CG	2.63 ± 0.49		0.94 (0.13)
RIQ	Pre-test	IG	2.03 ± 0.31	0.34 (0.73)	0.93 (0.06)
		CG	1.99 ± 0.48		0.94(0.11)
	Post-test	IG	1.48 ± 0.51	\	0.98 (0.85)
		CG	2.11 ± 0.59		0.95 (0.22)

IG, intervention group; CG, control group.

#### 3.3.2 Comparing baseline and post-intervention scores in intervention and control groups for depression, self-esteem, resilience, and relapse

**Figure 4:**
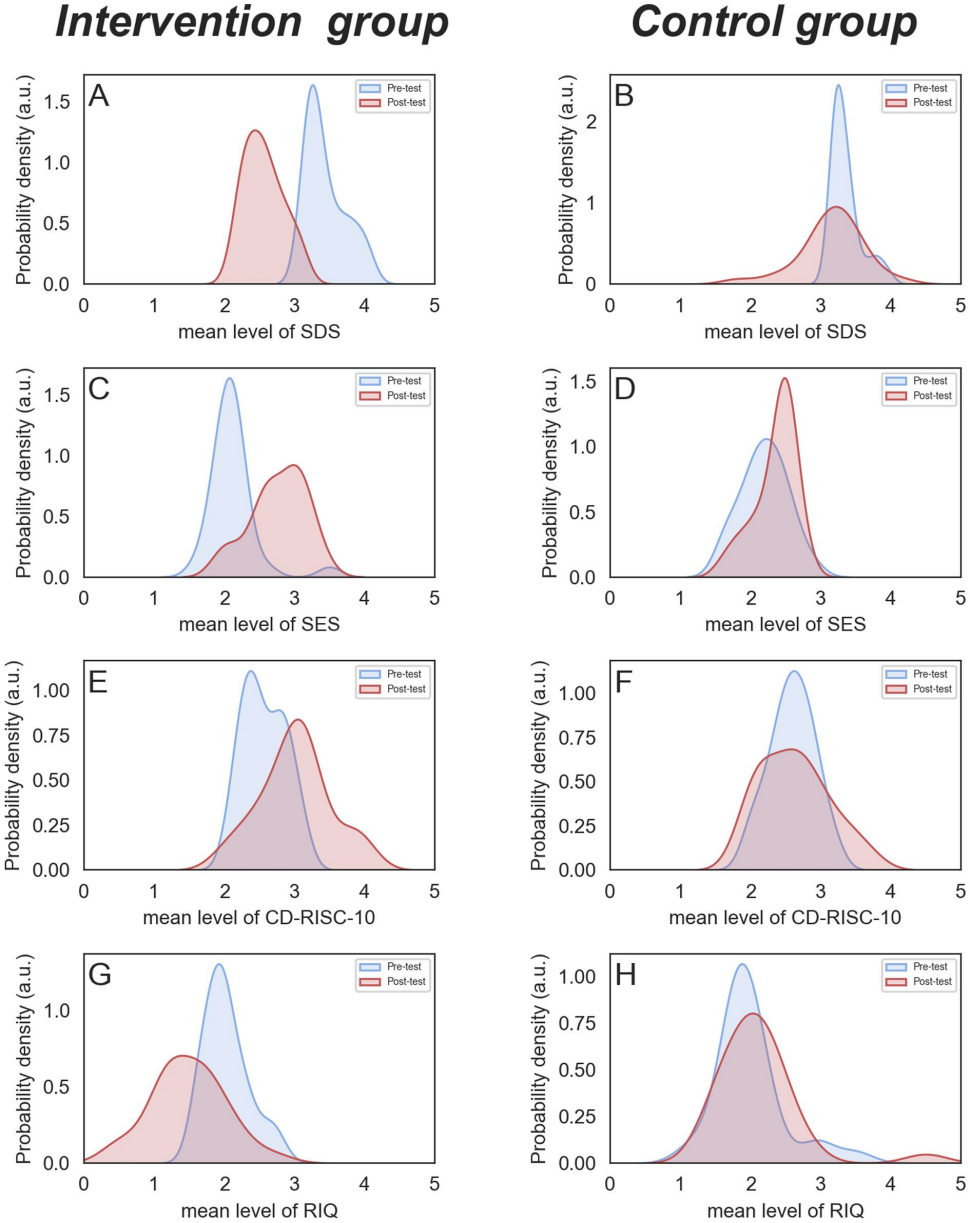
Pre-test and post-test comparison of SDS, SES, CD-RISC-10, and RIQ between the two groups.

The results of the two-factor mixed design ANOVA unveiled a notable interaction between group and time in SDS score (*F* = 36.92, *p* < 0.01, *partial η*^2^ = 0.40). Both the main effects of group (*F* = 16.70, *p* < 0.01, *partial η^2^* = 0.23) and time (*F* = 92.39, *p* < 0.01, *partial η^2^* = 0.63) were found to be statistically significant. The subsequent simple effect analysis indicated that the pre-test scores of the intervention group were higher than the post-test scores [*F* = 125.26, *p* < 0.01], while the control group’s scores remained relatively stable [*F* = 3.16, *p* = 0.08] (see Figures 4A,B).

Similarly, a significant interaction was observed between group and time for the SES score (*F* = 11.5, *p* < 0.01, *partial η^2^* = 0.17), with significant main effects for both group (*F* = 9.74, *p* < 0.01, *partial η^2^* = 0.15) and time (*F* = 32.45, *p* < 0.01, *partial η^2^* = 0.37). Simple effect analysis revealed an increase in the intervention group’s scores from pre-test to post-test [*F* = 43.50, *p* < 0.01], while the control group displayed no significant change [*F* = 2.5, *p* = 0.12] (see Figures 4C,D).

The analysis uncovered a statistically significant interaction between group and time in terms of the CD-RISC-10 score (*F* = 7.56, *p* < 0.01, *partial η^2^* = 0.12), with significant main effects for both group (*F* = 10.46, *p* < 0.01, *partial η^2^* = *0*.16) and the group (*F* = 5.06, *p* < 0.05, *partial η^2^* = 0.08) reaching significance. Simple effect analysis showed an increase in the intervention group’s scores from pre-test to post-test [*F* = 18.22, *p* < 0.01], while the control group’s scores remained relatively constant [*F* = 0.12, *p* = 0.74] (see Figures 4E,F).

Regarding the RIQ score, the interaction between group and time was also statistically significant (*F* = 14.27, *p* < 0.01, *partial η^2^* = 0.21), with significant main effects for both group (*F* = 9.86, *p* < 0.01, *partial η^2^* = 0.15) and time (*F* = 5.95, *p* < 0.05, *partial η^2^* = 0.10). Simple effect analysis indicated a decrease in the intervention group’s scores from pre-test to post-test [*F* = 19.67, *p* < 0.01], whereas control group’s scores showed little variation [*F* = 0.38, *p* = 0.35] (see Figures 4G,H).

## 4 Discussion

This research adopts a dual approach that involves both cross-sectional investigation and Pilot RCT to explore the factors influencing relapse in individuals with SUDs and to assess the efficacy of targeted intervention programs. In the Study 1, a cross-sectional survey serves as a pioneering effort to examine the interplay between depression, self-esteem, resilience, and relapse among those with SUDs. The findings reveal that depression not only has a direct effect on relapse but also elucidates the distinct and sequential roles that self-esteem and resilience play in mediating this relationship. A comprehensive understanding of the psychological factors that contribute to relapse vulnerability is essential for creating effective interventions. Building on these insights, the research advances to a pilot RCT in Study 2, focusing on depression. It introduces an innovative Health Qigong exercise regimen, designed in accordance with the principles of embodied cognition and aimed at addressing the physical manifestations of depression. The 12-week Health Qigong program not only alleviates depressive symptoms but also enhances self-esteem and psychological resilience, significantly reducing the risk of relapse. By merging traditional Chinese sports with Western theories of embodied cognition, this innovative approach offers non-pharmacological treatment alternatives for substance dependence, contributing fresh strategies and perspectives to the realm of drug rehabilitation. Simultaneously, it also establishes a theoretical framework for the scientific study of Chinese traditional sports.

### 4.1 Depression and relapse

This study confirms a noteworthy positive correlation between depressive mood and propensity for relapse in individuals with SUDs. Depressive mood correlates with neurotransmitter dysregulation, including serotonin and dopamine, which are crucial to the brain’s reward pathway and implicated in drug addiction. Such dysregulation may impair neurotransmitter system functioning, consequently increasing relapse risk (49). Furthermore, depressive mood can also precipitate social withdrawal and less engagement in social activities, resulting in reduced access to essential social support networks. Given the critical role of social support in preventing relapse, insufficient social support amplifies vulnerability to relapse triggers (50). In summary, the effective identification and remediation of depressive symptoms can significantly lower relapse risk, accordingly enhancing treatment outcomes and promoting long-term recovery.

### 4.2 The mediating role of self-esteem and resilience

This study further explored how depression contributed to relapse, shedding light on the potential mediating role of self-esteem, thereby supporting H1. Notably, a significant negative correlation emerged between depression and self-esteem among individuals SUDs. Baker’s cognitive triad model of depression suggests that individuals experiencing depression are often prone to negative thinking patterns, including distorted self-perception, pessimism about the environment, and anticipation of negative outcomes. These patterns contribute to a detrimental cognitive cycle that diminishes self-esteem (51). Furthermore, the research demonstrated an inverse relationship between self-esteem and relapse risk in individuals with SUDs. Self-esteem is closely linked to self-efficacy, the belief in one’s ability to execute tasks and reach goals. Bandura highlighted that self-efficacy influences decision-making, goal setting, exertion of effort, perseverance through challenges, and stress management strategies (52). Individuals harboring diminished self-efficacy are predisposed to doubt their capacity to avoid temptations and sustain sobriety, thereby increasing susceptibility to relapse. They often view themselves as lacking efficacious methods for navigating life’s stressors and emotional difficulties, thus more frequently resorting to substance use as a coping mechanism. Enhancing self-efficacy has proven to be a viable preventive measure against relapse, thereby offering empirical support for the connection between self-esteem and relapse risk (53).

This study supports Hypothesis 2 by demonstrating that psychological resilience mediates the relationship between depression and the tendency to relapse. When confronted with stressful circumstances, depressive individuals tend to succumb to negative and repetitive thought patterns. This, in turn, adversely impacts their behavior and coping mechanisms, consequently diminishing their psychological resilience. Moreover, Pfau and Russo emphasized that resilience results from the integrated operation of various biological systems, such as the neuroendocrine, immune, and central nervous systems. Depression has the potential to compromise these systems, thereby reducing an individual’s resilience and their capacity to manage depression effectively (54). Furthermore, the research elaborates a significant correlation between increasing resilience among individuals with SUDs and a reduced likelihood of relapse. The Elaboration Likelihood Model elucidates the relationship between mental resilience and relapse by distinguishing two cognitive pathways in decision-making: the central and peripheral routes. The central route is engaged through meticulous evaluation, utilized when an individual exhibits high motivation and cognitive capacity. In contrast, the peripheral route, which depends on cognitive shortcuts, is triggered by low motivation or capacity (55). During depressive episodes, individuals grappling with drug dependence may default to the peripheral route, thereby heightening the risk of relapse. Conversely, individuals with substantial psychological resilience prefer the central route, employing profound reflection and efficacious coping strategies to avert behavioral disorders and foster better adjustment (56, 57). These findings indicate the pivotal role of psychological resilience both in bolstering the decision-making process and mitigating the risk of relapse.

The present study also revealed that the relationship between depression and relapse of individuals with SUDs can be mediated sequentially by self-esteem and resilience, thereby lending support to H3. High self-esteem predisposes individuals to interpret life events positively, which helps sustain an optimistic outlook in the face of adversity. This positive cognitive assessment is critical for the adoption of efficacious coping strategies, subsequently strengthening psychological resilience. Research by Mäkikangas et al. (58) highlighted optimism and self-esteem as fundamental and enduring components of resilience over time, underscoring their importance in bolstering mental resilience. Shu et al. (59) further accentuate self-esteem as an indispensable internal determinant for developing psychological resilience, vital for enhancing adaptability and reducing vulnerability. Consequently, depression undermines resilience through the erosion of self-esteem, thereby increasing the likelihood of relapse.

### 4.3 Intervention effect of health Qigong

Embodied cognition underscores the physical basis of cognitive processes, arguing that abstract mental representations are anchored in bodily movements and sensory experiences (60). Within this theoretical framework, George Lakoff and Mark Johnson’s conceptual metaphor theory suggests that metaphors are a core cognitive tool, enabling individuals to understand the world by relating intangible concepts to tangible, directly perceivable ones (61). This theory illustrates those abstract notions, such as time, are comprehended through metaphorical connections to more concrete domains, such as space (62). Research indicates that expansive postures can boost creativity by fostering cognitive flexibility and transforming thought processes. In contrast, contractile postures are beneficial for tasks requiring definitive answers or solutions (63). Additionally, societal gender roles are conceptualized and categorized through metaphors of “soft” and “hard.” This metaphorical thinking activates brain memory’s modal information related to proprioception, hence influencing perceptions of softness and hardness, even in unrelated objects like sofas, showcasing the embodied impact on cognitive judgments (64).

Building on this foundation, the study developed a Health Qigong intervention specifically designed for depressive symptoms. It features upward stretching and expanding movements, contrasting with the typical physical manifestations of depression. Surveys conducted before and after the intervention revealed that regular participation in this program significantly reduced depressive symptoms, enhanced self-esteem, improved psychological resilience, and lowered the risk of relapse. These findings provide preliminary, yet indirect support for the embodied cognition theory, which posits that physical exercise profoundly influences cognitive functions and, in turn, modulates thought processes, judgments, attitudes, and emotional states (65). Further validation of these outcomes requires more direct and objective testing methods.

### 4.4 Limitations and prospects

Although this study systematically investigated the determinants and mechanisms of relapse among individuals with SUDs and developed effective relapse prevention strategies, it is subject to several limitations. First, the sample representativeness was limited to male SUD individuals from Guangxi, China, which may affect the generalizability of the results. Future research should expand the sample to include participants of different genders, ages, cultural backgrounds, and geographical locations to enhance the external validity of the findings. Second, the study was limited to an immediate evaluation following a 12-week intervention period, which may hinder our understanding of the long-term effects and durability of the intervention’s impact. Future research should incorporate extended follow-up assessments to thoroughly examine the sustained effects of Health Qigong on relapse rates in individuals with SUDs. Third, all measures in the study were based on questionnaires, which are susceptible to personal bias. Incorporating physiological and/or behavioral measures could enhance the objectivity and reliability of the findings. Fourth, due to practical constraints, we could not employ advanced motion capture equipment and specialized emotional assessment tools. This limitation hindered our capacity to evaluate changes in body posture and the relationship between participants’ emotional states and their physical postures. As a result, our ability to fully explore the impact of Health Qigong intervention on embodied cognition elements was compromised, affecting the comprehensive validation of embodied cognition theory. Future research should use more sophisticated technologies in less restrictive settings to further validate and expand our theoretical hypotheses.

## 5 Conclusion

The current research has established a significant correlation between depression and relapse in individuals with SUDs, wherein self-esteem and resilience serve as both individual and sequential mediators within this association. In addition, the 12-week Health Qigong training, specifically targeting the physical manifestation of depressive symptoms, not only effectively alleviated depression in individuals with SUDs, but also enhanced their self-esteem and mental resilience, and reducing their likelihood of relapse.

## Data Availability

The raw data supporting the conclusions of this article will be made available by the authors, without undue reservation.

## References

[1] Darcq E Kieffer BL. Opioid receptors: drivers to addiction? Nat Rev Neurosci. (2018) 19:499–514. doi: 10.1038/s41583-018-0028-x, PMID: 29934561

[2] Enoch M. The influence of gene–environment interactions on the development of alcoholism and drug dependence. Curr Psychiatry Rep. (2012) 14:150–8. doi: 10.1007/s11920-011-0252-9, PMID: 22367454 PMC3470472

[3] Charlson FJ Baxter AJ Cheng HG Shidhaye R Whiteford H. The burden of mental, neurological, and substance use disorders in China and India: a systematic analysis of community representative epidemiological studies. Lancet. (2016) 388:376–89. doi: 10.1016/s0140-6736(16)30590-6 27209143

[4] Brandon TH Vidrine JI Litvin EB. Relapse and relapse prevention. Annu Rev Clin Psychol. (2007) 3:257–84. doi: 10.1146/annurev.clinpsy.3.022806.091455 17716056

[5] Sun C Wang X Huang X Shao Y Ling APK Qi H. Sleep disorders as a prospective intervention target to prevent drug relapse. Public Health. (2022) 10:10. doi: 10.3389/fpubh.2022.1102115, PMID: 36684873 PMC9846318

[6] Hunt GE Malhi GS Lai HMX Cleary M. Prevalence of comorbid substance use in major depressive disorder in community and clinical settings, 1990–2019: systematic review and meta-analysis. J Affect Disord. (2020) 266:288–304. doi: 10.1016/j.jad.2020.01.141 32056890

[7] Lingford-Hughes A Nutt D. Neurobiology of addiction and implications for treatment. Br J Psychiatry. (2003) 182:97–100. doi: 10.1192/bjp.182.2.97 12562734

[8] Zellner M Watt DF Solms M Panksepp J. Affective neuroscientific and neuropsychoanalytic approaches to two intractable psychiatric problems: why depression feels so bad and what addicts really want. Neurosci Biobehav Rev. (2011) 35:2000–8. doi: 10.1016/j.neubiorev.2011.01.003, PMID: 21241736

[9] Conway KP Compton WM Stinson FS Grant BF. Lifetime comorbidity of DSM-IV mood and anxiety disorders and specific drug use disorders. J Clin Psychiatry. (2006) 67:247–58. doi: 10.4088/jcp.v67n0211 16566620

[10] Grant BF Saha TD Ruan WJ Goldstein RB Chou SP Jung J. Epidemiology ofDSM-5Drug use disorder. JAMA Psychiatry. (2016) 73:39–47. doi: 10.1001/jamapsychiatry.2015.2132, PMID: 26580136 PMC5062605

[11] Lai HMX Cleary M Sitharthan T Hunt GE. Prevalence of comorbid substance use, anxiety and mood disorders in epidemiological surveys, 1990–2014: a systematic review and meta-analysis. Drug Alcohol Depend. (2015) 154:1–13. doi: 10.1016/j.drugalcdep.2015.05.031, PMID: 26072219

[12] Grant BF Stinson FS Dawson DA Chou SP Dufour MC Compton WM. Prevalence and co-occurrence of substance use disorders and independent mood and anxiety disorders. Arch Gen Psychiatry. (2004) 61:807–16. doi: 10.1001/archpsyc.61.8.807, PMID: 15289279

[13] Newman SD. Association between hormonal birth control, substance use, and depression. Psychiatry. (2022) 13:13. doi: 10.3389/fpsyt.2022.772412, PMID: 35211041 PMC8861494

[14] Khantzian EJ. The self-medication hypothesis of addictive disorders: focus on heroin and cocaine dependence. Am J Psychiatry. (1985) 142:1259–64. Available from: doi: 10.1176/ajp.142.11.1259, PMID: 3904487

[15] Shahar G Davidson L. Depressive symptoms erode self-esteem in severe mental illness: a three-wave, cross-lagged study. J Consult Clin Psychol. (2003) 71:890–900. doi: 10.1037/0022-006x.71.5.890, PMID: 14516237

[16] Deng HH Chen H Zhong P Liang ZB Zhang GZ. Cross-lagged regression analysis of relationship between self-esteem and depression in early adolescents: test of vulnerability model and scar model. Psychol Dev Educ. (2013) 29:407–14. doi: 10.16187/j.cnki.issn1001-4918.2013.04.006

[17] Schiller M Hammen C Shahar G. Links among the self, stress, and psychological distress during emerging adulthood: comparing three theoretical models. Self Identity. (2016) 15:302–26. doi: 10.1080/15298868.2015.1131736

[18] Crowe E Daly M Delaney L Carroll S Malone K. The intra-day dynamics of affect, self-esteem, tiredness, and suicidality in major depression. Psychiatry Res. (2019) 279:98–108. doi: 10.1016/j.psychres.2018.02.032, PMID: 29661498

[19] Mruk CJ. Self-esteem and positive psychology: research, theory, and practice. Psychology. (2013) 50:50–7050. doi: 10.5860/choice.50-7050

[20] Yin SM Zhang F Shen MW Zhu HY Xu M. Constructing heroin dependent personality and its relation to mental health. Chin J Drug Depend. (2005) 14:112–6. doi: 10.13936/j.cnki.cjdd.1992.2005.02.011

[21] Swaim RC Stanley LR. Self-esteem, cultural identification, and substance use among American Indian youth. J Community Psychol. (2019) 47:1700–13. doi: 10.1002/jcop.22225, PMID: 31374591 PMC8201966

[22] Musyoka CM Mbwayo A Donovan DM Mathai M. Alcohol and substance use among first-year students at the University of Nairobi, Kenya: prevalence and patterns. PLoS One. (2020) 15:e0238170. doi: 10.1371/journal.pone.0238170, PMID: 32857791 PMC7454962

[23] Connor KM Davidson J. Development of a new resilience scale: the Connor-Davidson resilience scale (CD-RISC). Depress Anxiety. (2003) 18:76–82. doi: 10.1002/da.10113 12964174

[24] Richardson GE Neiger BL Jensen SE Kumpfer KL. The resiliency model. Health Educ. (1990) 21:33–9. doi: 10.1080/00970050.1990.10614589

[25] Waugh CE Koster EHW. A resilience framework for promoting stable remission from depression. Clin Psychol Rev. (2015) 41:49–60. doi: 10.1016/j.cpr.2014.05.004, PMID: 24930712

[26] Vesco AT Howard KR Anderson L Papadakis JL Hood KK Weissberg-Benchell J. Examining indirect effects of anxiety on glycated hemoglobin via automatic negative thinking and diabetes-specific distress in adolescents with type 1 diabetes. Can J Diabetes. (2021) 45:473–80. doi: 10.1016/j.jcjd.2021.05.002, PMID: 34176611 PMC8239251

[27] Yamashita A Yoshioka S Yajima Y. Resilience and related factors as predictors of relapse risk in patients with substance use disorder: a cross-sectional study. Subst Abuse Treat Prev Policy. (2021) 16:40. doi: 10.1186/s13011-021-00377-8, PMID: 33947412 PMC8097930

[28] Kennedy B Chen R Fang F Valdimarsdóttir U Montgomery S Larsson H. Low stress resilience in late adolescence and risk of smoking, high alcohol consumption and drug use later in life. J Epidemiol Community Health. (2019) 73:496–501. doi: 10.1136/jech-2018-211815, PMID: 30718261

[29] Lee J Joo EJ Choi K. Perceived stress and self-esteem mediate the effects of work-related stress on depression. Stress Health. (2013) 29:75–81. doi: 10.1002/smi.2428, PMID: 22610597

[30] Connell JP Spencer MB Aber JL. Educational risk and resilience in African-American youth: Context, self, action, and outcomes in school. Child Dev. (1994) 65:493–506. doi: 10.2307/1131398 8013236

[31] Wright MOD Masten AS Narayan AJ. Resilience processes in development: Four waves of research on positive adaptation in the context of adversity. In: Handbook of Resilience in Children. Boston, MA: Springer US (2012).

[32] Lee JH Nam SK Kim A Kim B Lee MY Lee SM. Resilience: a meta-analytic approach. J Couns Dev. (2013) 91:269–79. doi: 10.1002/j.1556-6676.2013.00095.x

[33] Arslan G. Psychological maltreatment, emotional and behavioral problems in adolescents: the mediating role of resilience and self-esteem. Child Abuse Negl. (2016) 52:200–9. doi: 10.1016/j.chiabu.2015.09.010 26518981

[34] Foulds J Adamson SJ Boden JM Williman J Mulder R. Depression in patients with alcohol use disorders: systematic review and meta-analysis of outcomes for independent and substance-induced disorders. J Affect Disord. (2015) 185:47–59. doi: 10.1016/j.jad.2015.06.024 26143404

[35] Huang J Zheng Y Gao D Hu M Yuan T. Effects of exercise on depression, anxiety, cognitive control, craving, physical fitness and quality of life in methamphetamine-dependent patients. Front Psychiatry. (2020) 10:10. doi: 10.3389/fpsyt.2019.00999, PMID: 32047445 PMC6997340

[36] Heyman E Gamelin FX Goekint M Piscitelli F Roelands B Leclair E. Intense exercise increases circulating endocannabinoid and BDNF levels in humans—possible implications for reward and depression. Psychoneuroendocrinology. (2012) 37:844–51. doi: 10.1016/j.psyneuen.2011.09.017, PMID: 22029953

[37] Liu F Cui J Liu X Chen KW Chen X Li R. The effect of tai chi and Qigong exercise on depression and anxiety of individuals with substance use disorders: a systematic review and meta-analysis. BMC Complement Med Ther. (2020) 20:161. doi: 10.1186/s12906-020-02967-8, PMID: 32471415 PMC7260819

[38] Anderson M. How to study the mind: an introduction to embodied cognition. Cambridge: Cambridge University Press (2005).

[39] Eriksson S Gard G. Physical exercise and depression. Phys Ther Rev. (2011) 16:261–8. doi: 10.1179/1743288x11y.0000000026

[40] Darwin C. The expression of the emotions in man and animals. London: John Murray (1872).

[41] Michalak J Troje NF Fischer J Vollmar P Heidenreich T Schulte D. Embodiment of sadness and depression—GAIT patterns associated with dysphoric mood. Psychosom Med. (2009) 71:580–7. doi: 10.1097/PSY.0b013e3181a2515c, PMID: 19414617

[42] Lemke MR Wendorff T Mieth B Buhl K Linnemann M. Spatiotemporal gait patterns during over ground locomotion in major depression compared with healthy controls. J Psychiatr Res. (2000) 34:277–83. doi: 10.1016/s0022-3956(00)00017-0, PMID: 11104839

[43] Osypiuk K Thompson E Wayne PM. Can tai chi and Qigong postures shape our mood? Toward an embodied cognition framework for mind-body research. Front Hum Neurosci. (2018) 12:12. doi: 10.3389/fnhum.2018.00174, PMID: 29765313 PMC5938610

[44] Gupta RK. Major depression: an illness with objective physical signs. World J Biol Psychiatry. (2009) 10:196–201. doi: 10.1080/15622970902812072 19396703

[45] Buyukdura JS McClintock SM Croarkin PE. Psychomotor retardation in depression: biological underpinnings, measurement, and treatment. Prog Neuropsychopharmacol Biol Psychiatry. (2011) 35:395–409. doi: 10.1016/j.pnpbp.2010.10.019, PMID: 21044654 PMC3646325

[46] Zung WWK. A self-rating depression scale. Arch Gen Psychiatry. (1965) 12:63. doi: 10.1001/archpsyc.1965.01720310065008 14221692

[47] Rosenberg M. Society and the adolescent self-image. Princeton: Princeton University Press (1965).

[48] Zhang ZA. A study of heroin addicts’ re-addiction tendency. J Psychol Sci. (2004) 27:739–40. doi: 10.16719/j.cnki.1671-6981.2004.03.061

[49] Belujon P Grace AA. Dopamine system dysregulation in major depressive disorders. Int J Neuropsychopharmacol. (2017) 20:1036–46. doi: 10.1093/ijnp/pyx056, PMID: 29106542 PMC5716179

[50] Wiss DA Prelip M Upchurch DM von Ehrenstein OS Tomiyama AJ Shoptaw S. Perceived social support moderates the association between household dysfunction adverse childhood experiences (ACEs) and self-reported drug use among men who have sex with men in Los Angeles, California. Int J Drug Policy. (2022) 110:103899. doi: 10.1016/j.drugpo.2022.103899, PMID: 36334318

[51] Beck AT. Depression, clinical, experimental, and theoretical aspects. Ann Intern Med. (1968) 68:502. doi: 10.7326/0003-4819-68-2-502

[52] Bandura A Freeman W Lightsey R. Self-efficacy: the exercise of control. J Cogn Psychother. (1999) 13:158–66. doi: 10.1891/0889-8391.13.2.158

[53] Marlatt GA Donovan DM. Relapse prevention: maintenance strategies in the treatment of addictive behaviors. 2nd ed. New York: Guilford Press (2005).

[54] Pfau ML Russo SJ. Peripheral and central mechanisms of stress resilience. Neurobiol Stress. (2015) 1:66–79. doi: 10.1016/j.ynstr.2014.09.004, PMID: 25506605 PMC4260357

[55] Lee MT Theokary C. The superstar social media influencer: exploiting linguistic style and emotional contagion over content? J Bus Res. (2021) 132:860–71. doi: 10.1016/j.jbusres.2020.11.014

[56] Witkiewitz K Marlatt GA. Relapse prevention for alcohol and drug problems: that was zen, this is tao. Am Psychol. (2004) 59:224–35. doi: 10.1037/0003-066x.59.4.224, PMID: 15149263

[57] Olsson CA Bond L Burns J Vella-Brodrick D Sawyer SM. Adolescent resilience: a concept analysis. J Adolesc. (2003) 26:1–11. doi: 10.1016/s0140-1971(02)00118-5 12550818

[58] Mäkikangas A Kinnunen U Feldt T. Self-esteem, dispositional optimism, and health: evidence from cross-lagged data on employees. J Res Pers. (2004) 38:556–75. doi: 10.1016/j.jrp.2004.02.001

[59] Shu YC Yang J Yang SY Wang YX Huang PF Lin WZ. The influence of social support on anxiety among university students during COVID-19 control phase: the chain mediating roles of self-esteem and resilience. Chin J Clin Psychol. (2021) 29:1333–6. doi: 10.16128/j.cnki.1005-3611.2021.06.043

[60] Barsalou LW. Grounded cognition. Annu Rev Psychol. (2008) 59:617–45. doi: 10.1146/annurev.psych.59.103006.093639 17705682

[61] Lakoff G Johnson M. Metaphors we live by. Chicago: University of Chicago Press (1980).

[62] Boroditsky L. Metaphoric structuring: understanding time through spatial metaphors. Cognition. (2000) 75:1–28. doi: 10.1016/s0010-0277(99)00073-6 10815775

[63] Michinov N Michinov E. Do open or closed postures boost creative performance? The effects of postural feedback on divergent and convergent thinking. Psychol Aesthet Creat Arts. (2022) 16:504–18. doi: 10.1037/aca0000306

[64] YI Z YANG W YE H. Influence of soft and hard tactical experiences on gender role cognition. Xin Li Xue Bao. (2018) 50:793–802. doi: 10.3724/SP.J.1041.2018.00793

[65] Niedenthal PM Winkielman P Mondillon L Vermeulen N. Embodiment of emotion concepts. J Pers Soc Psychol. (2009) 96:1120–36. doi: 10.1037/a0015574 19469591

