# Supplementary material for: Embodied cognition driven Qigong: a cross-sectional study and a pilot randomized controlled trial on managing depression and preventing relapse in substance dependence
Source: Front Public Health. 2024 Nov 1;12:1388887. doi: 10.3389/fpubh.2024.1388887 (PMC11564176; doi:10.3389/fpubh.2024.1388887)
Supplement: Supplementary file 1 [file Table_1.DOCX]

**Table1 Health Qigong Action Guidance Chart**

| **Action Name** | **Action Guide** |
| --- | --- |
| The ready form | Action One: Stand with feet together; tilt your head back slightly, keep your neck straight, lips lightly closed, and the tip of your tongue lightly touching the upper palate. Relax the area between your eyebrows and the corners of your mouth; let your arms hang naturally by your sides, with shoulders relaxed and elbows drooping. Keep your wrists loose, fingers relaxed, and the pads of your middle fingers lightly touching the seam of your trousers. Keep your armpits slightly closed, chest naturally expanded, and abdomen relaxed; look straight ahead.  Action Two: As you relax your waist and lower your hips, shift your body weight to your right leg and step out to the left with your left foot, about shoulder-width apart, toes pointing forward. Then, evenly distribute your weight between both legs; continue looking straight ahead.  Action Three: Rotate both arms inward, swing your palms out to the sides, with the arms forming a 45-degree angle with your body, and palms facing backward; maintain your gaze forward.  Action Four: Lower your body weight straight down, bending the knees of both legs; simultaneously, rotate both arms outward, bring your palms forward and up to a 45-degree angle in front of you, then bend your elbows and wrists to form a ball-holding position, with palms facing inward, at the same height as your navel, and fingertips of both hands facing each other, spaced 10 to 20 centimeters apart; continue looking straight ahead. |
| Holding sky with hands | Action One: Rotate both arms outward and lower them in front of the lower abdomen, palms facing up, with fingertips approximately 10 centimeters apart and the little finger side about 10 centimeters from the lower abdomen; gaze forward.  Action Two: Spread the fingers of both hands and cross them in front of the lower abdomen; continue looking straight ahead.  Action Three: Gradually raise the body's center of gravity; at the same time, bend both elbows and lift both palms vertically up to chest level, palms facing up; gaze forward.  Action Four: Slowly straighten both legs; simultaneously, rotate both arms inward, lifting the palms upward with slightly bent elbows, palms facing up; lift your head and look at your palms.  Action Five: Continue to lift both palms, straightening the elbow joints; simultaneously, tuck in the chin; pause briefly, keeping the arms stretched; look straight ahead.  Action Six: Slowly lower the body's center of gravity, bending the knees; simultaneously, slowly separate the fingers, and as both arms lower to about a 45-degree angle to the sides of the body, bend the elbows and hold the palms in front of the abdomen, palms facing up, fingertips about 10 centimeters apart; gaze forward. |
| Shuangyu xuange | Action One: As you inhale, lift and contract your pelvic muscles, pulling in your abdomen. Turn your body to the left and straighten both legs. At the same time, rotate both arms inward and swing both palms sideways, stretching the arms with palms slightly lower than the shoulders and facing backwards; look straight ahead to the left front. As you exhale, relax the abdomen and pelvic muscles, turn your body to the right, shifting your weight to the right foot. Semi-squat on your right leg while lifting the heel of your left foot into a left T-step. At the same time, rotate your left arm outward, drawing your left palm in front of the lower right abdomen, palm facing up; draw your right palm inward and downward to rest above your left wrist, with the pad of the ring finger at the Taiyuan acupoint in a pulse-checking position; glance at your hand with the corner of your eye.  Action Two: As you inhale, lift and contract your pelvic muscles, pulling in your abdomen; turn your body to the left, stepping forward with your left foot from an empty stance to a bow stance. Meanwhile, maintain the pulse-checking position with both hands, swinging them in an arc forward to the left front of your body, with your left arm naturally straightened and the palm facing up; look at both palms. As you exhale, shift your weight backward, turning your body to the right into a left empty stance with the tip of your left foot raised. At the same time, rotate your left arm inward and your right arm outward, twisting your right palm fingers to touch the Taiyuan acupoint behind, overlapping with the left palm in front of the chest, palms facing each other, about 20 cm from the chest, left palm facing outward; glance at both palms with the corner of your eye.  Action Three: As you inhale, lift and contract your pelvic muscles, pulling in your abdomen; step your left foot next to your right, gradually straightening both legs. At the same time, slightly rub both palms horizontally against each other, then rotate your left arm inward, pressing your left palm next to your left hip, about 20 cm from the hip, forming an arc with your left arm, left palm pointing to the right; your right palm follows the inward rotation of your right arm, raised above the right front of your head, right arm forming an arc, right palm pointing left; look left horizontally.  Action Four: As you exhale, relax your abdomen and pelvic muscles; at the same time, keep your left hand still while your right palm follows your right arm, pressing slightly downward and forward to the right; shift your gaze to your right palm. Continue the movement without pause, allowing your right palm to fall alongside your left, both hanging naturally at your sides in a standing position; gaze forward as usual. |
| Longdeng | Action one: Stand with your heels as the pivot, toes pointed outward in a V-shape. Slowly lift your palms to the sides of your waist, palms slanted upward; gaze forward.  Action two: Bend your knees to squat; at the same time, thrust both palms diagonally forward and downward, visualizing the descent of turbid energy. When fully squatting, turn your palms upward, presenting them in front of the chest in a lotus shape; look at your palms.  Action three: Rise to stand straight, slowly raising your palms above your head; gaze upward and forward.  Action four: Spread your palms outward from the wrists, fingertips pointing outward; at the same time, slowly lift your heels; gaze downward and forward.  Action five: Lower your heels, bring your palms together below your chest and press downward, fingertips facing each other, then rotate your arms outward, flipping your palms; spread your shoulders outward, with your middle fingers pressing on the Dabao acupoint; look forward. At the end, let your palms naturally fall to the sides of your body; gaze forward. |
| Tiger pounce | Action one: Clasp your hands into empty fists and lift them along the sides of your body to chest level; keep the lower limbs stationary and slightly lean back the body.  Action two: Raise both hands upward and forward in an arc, then immediately bend the fingers into tiger claws, palms facing downward; simultaneously lean forward from the upper body, arching the back and sticking out the chest; glare forward angrily.  Action three: Bend your knees to squat, pulling in the abdomen and chest; at the same time, draw both hands downward in an arc to the sides of the knees, palms facing down; look downward and forward. Then, straighten your knees, push your hips forward, protrude your abdomen, lean back, forming an arch with your body; simultaneously, clasp your hands into empty fists and lift them along your body sides to chest level; gaze upward and forward.  Action four: Pivot the tip of your right foot outward by about 30°, shifting your weight to the right leg, lift the left foot, and continue moving both hands upward and forward in an arc, then step forward with the left foot, landing on the heel, bending the right knee into a squat to form a left empty stance; simultaneously lean the upper body forward about 45°, both fists turn into tiger claws and press down forward to the sides of the knees, level with the knees, the hands about two shoulder-widths apart, palms facing down; glare downward and forward. Then retract the left foot to shoulder width, bending both knees; draw both hands downward in an arc to the sides of the knees; look downward and forward. Finally, raise both palms forward to the sides of the body, palms facing up, level with the chest, then bend both arms at the elbows, palms coming together, turning the palms inward toward the Danzhong acupoint, then rotate the palms inward and slowly press down in front of the abdomen, separating to the left and right, arms hanging at the sides of the body; gaze forward. |
| Rouji style | Action one: Shift your weight to the left, drawing the right foot close to the inside of the left foot with the right foot's sole on the ground, forming a right T-step; simultaneously, swing both arms downward, to the left, and upward. When the left arm reaches shoulder height, the palm faces down and the fingertips point left; the right arm swings down to the left at about 45°, fingertips pointing left. Slightly bend both elbows, inhaling, and gaze at the left hand.  Action two: Continue the movement. The left leg remains bent at about 45°, the right foot pivots on its sole, moving the right leg outward until the toes point right; simultaneously, swing the arms to the left, the torso bends to the right side, tilting about 45° to the right, moving the left arm upward and to the right to a position 45° to the upper right, with a slight bend in the elbow, palm facing up, and fingertips pointing right. The right hand moves under the left armpit, the Laogong acupoint of the right hand aligning with the Dabao acupoint, 10 centimeters apart, elbows slightly bent and armpits hollow. Exhale in coordination, follow the left hand with your eyes, turn the head to the right as the torso bends about 45° to the right, pause slightly, and look downward to the right.  Action three: From the final pose of Action 2, follow the original movement path back to Action 1.  Action four: Step the right foot to the right, slightly wider than shoulder-width, shift weight to the right, slightly bend the right knee, retract the left foot to the inside of the right foot, sole of the left foot on the ground, forming a T-step; simultaneously, swing both arms downward and to the right, the right arm reaching shoulder height, palm facing down, fingertips pointing right; the left arm swings to the lower right, about 45° below the horizontal, slightly bend both elbows, palms facing down, fingertips pointing outward. Inhale in coordination, gaze at the right hand. Then, encircle both arms upward, fingertips opposite each other about 10 centimeters apart, palms facing down, arms forming an arc; inhale in coordination, gaze forward. Then, bend both legs about 45°; at the same time, draw breath back to the origin, press both palms down in front of the abdomen, level with the navel, about 10 centimeters apart, fingertips diagonally opposite each other; exhale in coordination, gaze downward and forward. |
| Deer running | Action one: Let your arms hang naturally at your sides; look straight ahead.  Action two: Rotate both arms inward and thrust them downward and forward, making about a 30-degree angle with your body; at the same time, arch your back and raise your heels. When arching your back, look at the tips of your index fingers on both hands.  Action three: Following the momentum, lower your heels, shift your weight to the right, and turn your body 45 degrees to the left, stepping forward with your left foot; simultaneously, rotate both arms outward and raise them, with the backs of the hands brushing against the ribs; look toward the front left.  Action four: Shift your weight forward, swing both arms up along the sides of your body in an arc, backs of the hands facing each other, forming hooked hands at shoulder height; raise the right heel and look at both palms. Shift your weight backward, sit back with your body, and let the right heel fall naturally; turn both palms outward, slightly bend the wrists, and extend the arms while arching the back; look at the wrists facing each other.  Action five: Shift your weight forward, lift the right heel naturally, let both palms fall and press down at the sides of your body; stretch up from the top of your head, looking into the distance.  Action six: Retract your left foot, turn your body to face forward, and let both arms hang naturally at your sides; look straight ahead. Finally, pull the right foot back to stand with feet together; look straight ahead. |
| Golden rooster dawn | Action one: Following the previous position, step left with the left foot, toes pointing forward, feet slightly wider than shoulder-width, and knees naturally straight; at the same time, raise both arms to the sides until they are level, with elbows slightly bent, palms facing up, and fingertips pointing outward; inhale and look forward.  Action two: Bend your knees to squat about 45 degrees; simultaneously, lift your head and tail up, arch your spine, lower your shoulders and elbows, and flare your wrists, with palms facing up, the base of the palms level with your ears, fingertips pointing outward; exhale, pause the motion slightly, and gaze upward and forward.  Action three: Straighten both knees; at the same time, retract your chin, keep your head straight, tailbone hanging down, straighten your torso, and spread both arms to the sides, elbows slightly bent, palms facing up, fingertips pointing outward; inhale and look forward.  Action four: Shift your weight to the right, retract the left foot to step together, both knees straight; simultaneously, circle both arms upward, fingertips opposite each other, palms slanting downward; inhale and look forward. Then, draw the breath back to the origin, press both palms downward in front of the body to the height of the navel, 10 centimeters apart, fingertips diagonally facing each other; simultaneously, bend your knees to squat about 45 degrees; exhale and look downward and forward. |
| Bird flying | Action one: Slightly bend both legs; bring your palms together in front of your abdomen, spread your palms and stretch your fingers, with the palms slightly tilted upward and fingertips facing each other, about 5 cm apart; gaze downward in front. Straighten the right leg to stand on it alone, bend the left knee and lift the left leg, letting the calf hang naturally, toes pointing down; at the same time, raise both arms to the sides of your body to shoulder level, forming “bird wings” with palms facing down, level with the ears; look forward.  Action two: Slightly bend the right leg, drop the left foot next to the right foot, about half a shoulder-width apart, toes touching the ground, bend the knees to squat, forming a left T-step; simultaneously, drop both palms to the sides and bring them together in front of the abdomen, spread the palms and stretch the fingers, with the palms slightly tilted upward and fingertips facing each other, about 5 cm apart; gaze downward in front.  Action three: Straighten the right leg to stand on it alone, bend the left knee and lift the left leg, letting the calf hang naturally, toes pointing down; simultaneously, lift both palms along the sides of your body above your head, backs of the hands facing each other, about 5 cm apart, fingertips slanting upward; look forward.  Action four: Drop the left foot next to the right foot, shoulder-width apart, bend the knees to squat; simultaneously, drop both palms to the sides and bring them together in front of the abdomen, spread the palms and stretch the fingers, with the palms slightly tilted upward and fingertips facing each other, about 5 cm apart; gaze downward in front. Repeat actions one to four once. Then, let the right foot drop, stand with feet spread shoulder-width apart; let both arms hang naturally at your sides, looking forward. Finally, raise both palms forward to the sides of your body, palms facing up, level with the chest, then bend both arms at the elbows, palms coming together, turn the palms inward facing the Danzhong acupoint, then rotate the palms inward and slowly press down in front of the abdomen, separating to the left and right, arms hanging at the sides of the body; look forward. |
| Returning qi to the source | Action one: Raise both palms along the sides of your body, palms facing up, lifting above the head, then turn the palms downward, diagonally facing the Baihui acupoint; look forward.  Action two: With fingertips of both palms facing each other, slowly press down in front of the body, separate the palms in front of the abdomen, and let both arms hang at the sides of the body; look forward.  Action three: Draw both palms back in a flat arc, level with the navel, palms facing backward, then rotate both arms outward, turning the palms forward; look forward.  Action four: Continue moving both palms forward in a flat arc, bringing them together in front of the abdomen, with the bases of the thumbs crossing, males with the left hand inside, females with the right hand inside, stacked in front of the navel; close your eyes and rest quietly.  Action five: Slowly open your eyes, rub your palms together in front of your chest until warm.  Action six: Place the palms on the face, rub up and down around the face, bathing the face 3 to 5 times.  Action seven: Move both palms back along the top of the head, behind the ears, and down in front of the chest, letting both arms hang at the sides; look forward.  Action eight: Lift the left foot and bring it close to the right foot, touching the ground with the front of the foot first, then placing the whole foot firmly, returning to the ready position; look forward. |
| The concluding form | Action one: As you inhale, lift the anus and contract the abdomen; simultaneously, both palms rotate inward then outward, moving to the sides of the body, with the palms turning from facing backward to forward, and the arms forming an angle of about 60 degrees with the upper body, arms naturally straightened; eyes looking straight ahead.  Action two: As you exhale, relax the abdomen and anus; straighten both legs; at the same time, draw both palms inward to embrace and stack over the Guanyuan acupoint, with the left hand inside for males and the right hand inside for females; eyes gently closed.  Action three: After completing, let both palms hang at the sides of the body, slowly conclude the movements, ending the entire set of exercises. |
